# Age of enlightenment: long-term effects of outdoor aesthetic lights on bats in churches

**DOI:** 10.1098/rsos.161077

**Published:** 2017-08-09

**Authors:** Jens Rydell, Johan Eklöf, Sonia Sánchez-Navarro

**Affiliations:** 1 Department of Biology, Lund University, Lund, Sweden; 2 Graptolit Ord och Natur, Bollebygd, Sweden; 3 Estación Biológica de Doñana-CSIC, Seville, Spain

**Keywords:** global change, energy, cultural heritage, historic buildings, biodiversity, light pollution

## Abstract

We surveyed 110 country churches in south-western Sweden for presence of brown long-eared bats *Plecotus auritus* in summer 2016 by visual inspection and/or evening emergence counts. Each church was also classified according to the presence and amount of aesthetic directional lights (flood-lights) aimed on its walls and tower from the outside. Sixty-one of the churches had previously been surveyed by one of us (J.R.) between 1980 and 1990, before lights were installed on Swedish churches, using the same methods. Churches with bat colonies had decreased significantly in frequency from 61% in 1980s to 38% by 2016. All abandoned churches had been fitted with flood-lights in the period between the two surveys. The loss of bat colonies from lit churches was highly significant and most obvious when lights were applied from all directions, leaving no dark corridor for the bats to leave and return to the roost. In contrast, in churches that were not lit, all of 13 bat colonies remained after 25+ years between the surveys. Lighting of churches and other historical buildings is a serious threat to the long-term survival and reproduction of light-averse bats such as *Plecotus* spp. and other slow-flying species. Bat roosts are strictly protected according to the EU Habitats Directive and the EUROBATS agreement. Lighting of buildings for aesthetic purposes is becoming a serious environmental issue, because important bat roosts are destroyed in large numbers, and the problem should be handled accordingly. As a start, installation of flood-lights on historical buildings should at least require an environmental impact assessment (EIA).

## 1. Introduction

Artificial illumination spreads rapidly throughout much of the world at present and this has an increasingly detrimental effect on nocturnal wildlife [1], including bats [2] and probably diurnal life-forms such as humans as well [3]. Over hundreds of millions of years, organisms have become adapted to the daily light--dark cycle through evolution of circadian rhythms, which influence the basic life patterns from physiology and reproduction to ecology and evolution [4]. For nocturnal animals such as most bats, darkness is also important in itself because it provides the principal protection against predation and therefore influences the survival and reproduction directly [5,6]. However, the relationship between bats and light is complicated, and the bats' response to light depends on the species [7] and prevailing conditions [8], the type of light [9], presence of predators [10] and what the bat is doing, e.g. emerging from roost, commuting, feeding or drinking [11–14].

Bats typically live many years, reproduce slowly and are adapted to low mortality rates, which means that their ability to compensate for increased predation is limited [15]. Regardless of species of bat, protection by darkness is particularly important at maternity roosts, places that often are used more or less consistently over long periods of time and where many individuals predictably pass every night on their way to and from the feeding sites. Maternity roosts are also places where the young learn to fly and where sit-and-wait predators like owls and cats may be a serious threat [16]. Illumination of buildings where bats reside exposes the bats to increased predation risk and the bats change their behaviour accordingly. For example, activity patterns and movements are disrupted, which in turn result in deteriorating foraging opportunities, lower food consumption and ultimately slower growth and lower survival particularly of the young [17–19]. In some cases strong lights may even inhibit the feeding flights entirely and abruptly, which may lead to starvation and finally extinction of the entire bat colony [20].

In northern Europe colonies of brown long-eared bats *Plecotus auritus* (Linné, 1758) typically reside in roof spaces of old buildings, and actively select those with large attics and several compartments [21] such as churches. Indeed, towers and attics of churches seem to provide particularly suitable summer roosts for colonies of this species in Sweden and other northern areas [22].

Bats as well as their roosts are strictly protected according to the EU Habitats Directive (Council Directive 92/43/EEC) and its national implementations, and the EUROBATS agreement (www.eurobats.org) should provide further protection. Nevertheless, the potentially devastating effect of lights on bat colonies in churches and other buildings has so far received little attention from the conservation point of view as well as from researchers.

Given the potential impact of exterior lighting installations on the persistence of bat colonies in churches, we undertook a study to examine the following specific questions: (i) Are long-eared bat colonies less frequent in flood-lit churches compared to unlit churches? (ii) Are long-eared bat colonies less frequent in churches that were once dark (unlit), but subsequently lit, compared to churches that have remained dark over time?

As far as we know this is the first study of the long-term consequences of installations of aesthetic lights on buildings with bat colonies.

## 2. Material and methods

### 2.1. Time of study and selection of churches

The first part of this study was made in the summers 1980–1990, when attics and towers of 61 country churches in south-western Sweden (Västra Götaland province, Skara diocese) were inspected by one of us (J.R.). The selection of churches was usually made without any prior knowledge of presence of bats, but in some cases churches were visited following telephone calls from wardens who had found bats. Hence, the selection of churches surveyed was slightly biased and the observed frequency of bat occurrence was higher than it would be in an entirely random sample. The same churches were again surveyed between May and early October 2016, using the same method. We then added another 48 churches, in this case without any prior information on bat presence, bringing the total number of surveyed churches to 110. Comparisons over time were made using only the 61 churches that were visited in both periods.

Our observations suggest that long-eared bat colonies in the study area normally remain in the churches at least between May and early October and we implicitly assumed that the likelihood of detecting the bats was the same throughout the survey periods. However, in reality the chance of detection may have increased somewhat in late summer as the young were born and droppings and food remains accumulated. However, we believe that the effect of such a seasonal bias on our results is small.

### 2.2. Habitat description

The origin of the oldest churches included in this survey goes back to AD 1100--1200, while the youngest were built during the nineteenth or early twentieth century. In the latter case larger churches were usually built to replace the smaller medieval ones. Relatively few churches were built AD 1400--1700, although many old ones were rebuilt and enlarged in this period. Although all the churches surveyed are relatively small (room for 50–250 people) and located in rural areas, they still vary in size, architecture, building material, roofing, setting and physical factors such as e.g. temperature and humidity under the roof. Also, in most but not all cases the churches were surrounded by a churchyard with mature deciduous trees. We do not include all this variation here, however, but concentrate on the lighting, which we believe is most relevant for our case. More importantly, the key part of this study is a pairwise (before--after) comparison of a set of churches over time, and it seems likely that most of the potentially confounding variation (except the lighting and perhaps also effects of renovations, see below) remained constant or nearly constant over the 25+ years study period.

**Figure 1.**
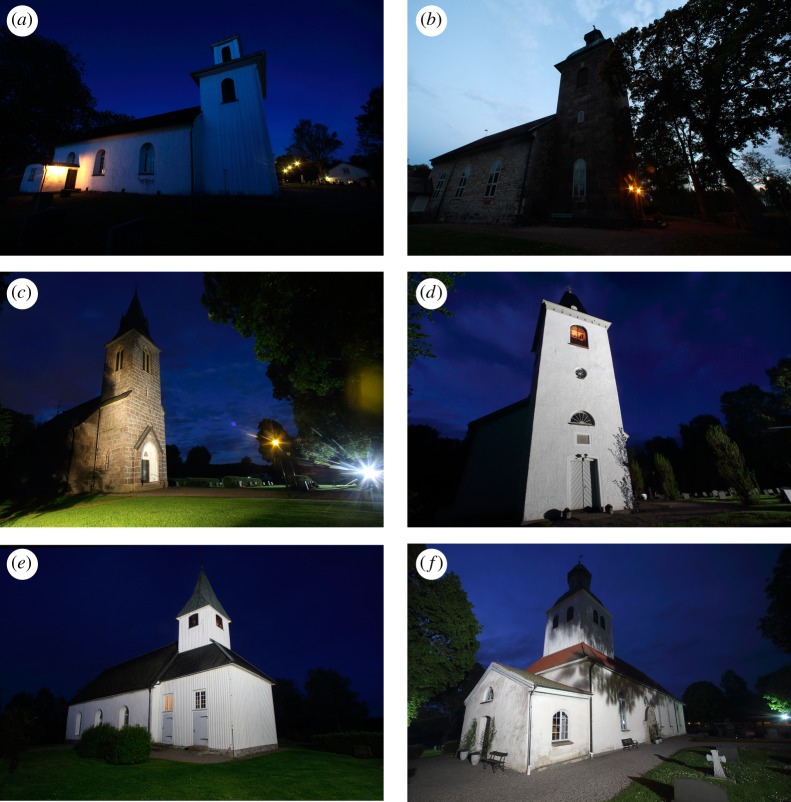
Examples of the three categories of flood-lighting used on the churches examined during this survey; (*a*,*b*) not lit, (*c*,*d*) partly lit, (*e*,*f*) fully lit. All churches shown harboured colonies of long-eared bats in the 1980s. In 2016 the colonies were gone from (*d*) and (*e*) but remained in (*a*–*c*) and (*f*).

We classified each church according to the presence/absence of aesthetic directional lights (flood-lights, directed more or less upwards) and the number and directions (1–4) from which these lights were aimed towards the walls and tower. We did not consider the intensity and colour of the lights, the lighting regime (i.e. part-time lighting) and if the lights were actually functioning at the time of the survey, as these aspects may have changed during the course of the study in a way that we could not control. Weaker non-directional lights, i.e. lamp-posts, were installed outside almost every church, alone or in addition to flood-lights. However, we did not consider such lights, as we believe they have much less impact on the bats than the stronger and more directional flood-lights. Safety lights, i.e. lights directed downwards from a high position, were not used at any of the churches. Some typical examples of churches and lighting included in the survey are shown in figure 1.

### 2.3. Roost inspection

The tower and attic of each church were inspected from the inside in daytime, using torches. Bats were scored according to the following criteria (i) no bats and no fresh droppings, (ii) fresh droppings and usually also prey remains (Lepidoptera wings), suggesting use by one or several long-eared bats as night-roost or feeding site, or regular use by several individuals that could not be seen, and (iii) direct observation of several adult and/or young individuals, confirming the presence of a maternity colony. In some cases during the 1980s survey, presence of bat colonies was inferred from large amounts of fresh droppings and other remains occurring in several places throughout the roost. In all cases bats observed inside the churches were brown long-eared bats *Plecotus auritus*. Lepidoptera wings were found in nearly every identified roost and indicated that it was occupied by *P. auritus* even in cases where no bats were seen and this was further confirmed by the size, texture and colour of droppings containing abundant Lepidoptera scales [23,24]. To minimize the disturbance and speed up the process, we did not attempt accurate counts of the bats from the inside, but only made minimum estimates based on bats immediately visible.

We are confident that our observations did not include species other than *P. auritus*. Other species that may feed extensively on Lepidoptera and possibly use regular feeding-perches, like those of *P. auritus*, are the grey long-eared bat *Plecotus austriacus*, Natterer's bat *Myotis nattereri*, Bechstein's bat *M. bechsteinii* and possibly the barbastelle *Barbastella barbastellus*, but none of these species occur in the study area as far as we know (J.R. 2017, unpublished data). However, northern bats *Eptesicus nilssonii* and soprano pipistrelles *Pipistrellus pygmaeus* or their remains were occasionally observed in the walls of churches from the outside, but we never detected any of these species from inside.

### 2.4. Recordings and emergence counts

In some cases during the 2016 survey, if droppings and food remains indicated presence of *P. auritus* although bats could not be seen, we left one or two automatic ultrasonic detectors (Pettersson D500X) inside the roost over night to confirm if bats were present. The recordings were later analysed using Pettersson BatSound version 4.03 and *P. auritus* was recognized from its typical relatively loud low frequency sweeps [25]. In such cases and when we could not get access to the relevant roof spaces, we also made an evening observation of emerging bats from the outside. One person was positioned on each side of the church and aided by a hand-held bat detector (Pettersson D960X and D1000X). Hence, presence of *P. auritus* colonies and the colony size were sometimes confirmed through observations from the outside. In all cases the emerging bats were unambiguously recognized as *Plecotus* because their diagnostic long ears could be seen.

### 2.5. Renovation history

To examine the potentially confounding effect of renovations on persistence of bat colonies, we tracked the history of 60 churches surveyed both in the 1980s and in 2016 (one church was excluded because it had lights installed inside the attic and therefore no longer qualified as a potential bat roost). We used information obtained from parish offices, church wardens and Internet sources (e.g. www.svenskakyrkan.se) in addition to our own notes from the surveys. We only considered major renovation work that affected the space used by bats in summer, i.e. the roof, attic and/or tower. We ignored normal repair work such as painting and minor wood work and also renovations that were restricted to other parts of the church. We compared the fate of the bat colonies with respect to renovation and the presence/absence of flood-lighting at each church (table 1).

**Table 1.** Summary of the occurrence of long-eared bat colonies in 60 churches surveyed in the 1980s and again in 2016. Effects of renovations and light installations are indicated by changes in the frequency of bat colony presence between the two surveys, i.e. colonies remained in the church, disappeared from it or were added to it.

	number of churches								
	unlit churches				flood-lit churches				
		bat colony				bat colony			
category	no bat colony	remaining	gone	added	no bat colony	remaining	gone	added	*N*
renovated	2	2	0	0	3	7	5	1	20
not renovated	9	11	0	0	9	3	8*	0*	40
total	11	13	0	0	12	10	13	1	60

* The decline in frequency of bat colonies among the non-renovated, lit churches is significant (*p* = 0.013).

For the statistical calculations we used the free Internet source www.graphpad.com.

## 3. Results

### 3.1. The 2016 survey

Brown long-eared bats used 64 (58%) of the 110 churches surveyed in 2016, as indicated either by live bats or fresh droppings and prey remains. In 40 churches (36%) presence of a colony was actually confirmed, as bats were seen either from inside the attic and/or tower in daytime or from the outside in the evening.

**Figure 2.**
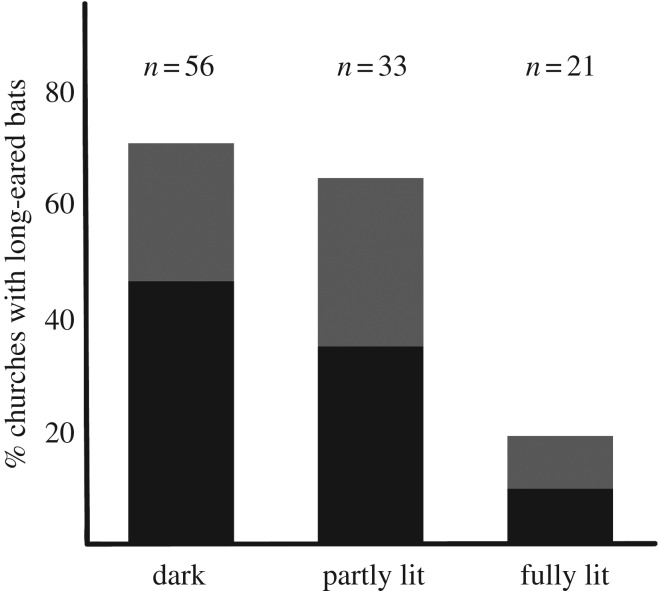
Result of the 2016 survey (*N* = 110 churches). The bars show the frequency of occurrence of long-eared bats or their remains in relation to the amount of flood-lighting on the church walls. The churches were either dark (unlit), partly lit or fully lit (lit from all directions). Black represents churches where colonies were actually observed.

Colonies and/or their fresh remains occurred significantly less frequently in churches lit by flood-lights compared to churches that were not lit (*p* < 0.05, Fisher's exact test, two-tailed). The effect was most obvious for churches that were lit from all sides, with no remaining dark corridor for bats to emerge and return (figure 2). Comparing such churches with those that were at least partly unlit, the difference in frequency of bat occurrence was highly significant (*p* < 0.0001, Fisher's exact test, two-tailed). Indeed, there were only two fully lit churches in which bat colonies were confirmed and circumstantial evidence suggests that these were special cases. At one of them, the flood-lights had actually been non-functional and disused over the past 8–10 years according to the warden, and at the other, the tower turned out to be shaded (figure 1*f*) and also, the lights were consistently turned off relatively early in the evening (23.00 h). Hence, there was not a single church that was fully lit for most of the night and where a *P. auritus* colony was confirmed.

### 3.2. Comparison with 1980s survey

Of the 61 churches surveyed in the 1980s, 46 (75%) showed live bats and/or prey remains from *P. auritus* and in 37 churches (61%) colonies occurred. When the same churches were surveyed again in 2016, the frequencies had decreased to 32 (52%) and 23 (38%), respectively. Over the 25+ years between the two surveys, 14 of the 24 original colonies that lived in churches that were subsequently lit had disappeared and one new colony was established in a (partly) lit church. This change in the frequency of bat presence over time is highly significant (*p* = 0.0019, McNemar's test with continuity correction, two-tailed). The result remains the same if all churches with prey remains (not only those with colonies) are included in the analysis.

**Figure 3.**
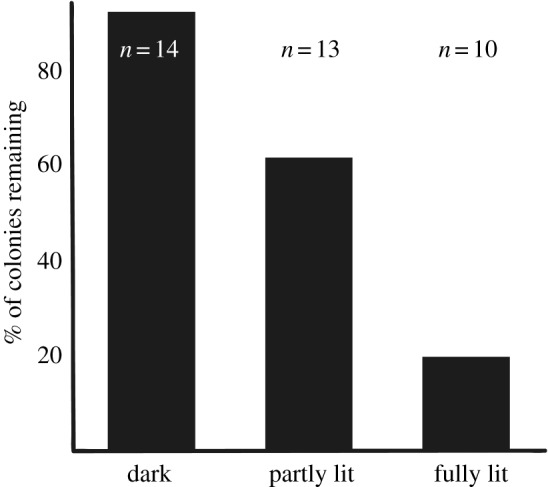
Result of the 1980s versus 2016 survey comparison (*N* = 37 churches that had colonies of long-eared bats in the 1980s). The bars represent churches where the colonies remained between the two surveys, i.e. after installations of flood-lights on some of them. The churches were either dark (unlit), partly lit or fully lit (lit from all directions).

The net loss between the two surveys was 13 colonies or 35%. The entire loss was from lit churches (figure 3). In contrast, 14 of the 37 churches that had colonies in the 1980s were still without flood-lights in 2016, and the bat colonies remained in all of them except one. The exception was a church where lights had been installed inside the attic (but with no lights from the outside) to display some interesting architecture to visitors.

### 3.3. Colony size

Minimum counts of bats inside 32 churches in the 1980s revealed 1–30 adults (median = 5), and counts in 23 churches in 2016 revealed 2–30 (median = 4). The emergence counts at six churches in 2016 was 2–32 adults (median = 5). The counts are almost certainly underestimates in many cases but nevertheless fall within the normal range for this species [24].

### 3.4. Effect of renovations

Twenty of the 60 churches examined had been subject to major renovations in attics and/or towers during the 25+ years between the two surveys, while 40 had not been affected in this way. The presence of bat colonies and the corresponding changes over time in lit and unlit churches and with respect to the renovation history is summarized in table 1. The data in the table indicate that: (i) Two of the unlit churches had been renovated and the bat colonies remained in both. Hence, the unlit churches do not provide any evidence that bats disappeared because of renovations. (ii) There was a significant decline in the number of bat colonies among the lit churches that were not renovated (*p* = 0.013; McNemar's test with continuity correction, two-tailed) but there was no significant decline among the renovated churches. Again, this indicates that churches were abandoned because of the lights rather than renovations. (iii) In four cases, bat colonies disappeared from churches that were both flood-lit and renovated. In these specific cases we cannot exclude the possibility that renovations were responsible to some extent. In summary, the disappearance of long-eared bat colonies from churches was most likely a result of light installations and not of renovations, at least in most cases.

## 4. Discussion

During our evening emergence counts we observed that the bats consistently appeared from a dark spot on the church and flew the shortest way to nearby tree cover. If the distance to cover was more than a few metres, the bats always flew straight and close (less than 1 m) to the church wall and the ground. We never saw a long-eared bat passing through or even near a light beam. It was obvious that they carefully avoided such exposure.

Some species of bats are more sensitive to lights than others and the vulnerability relates primarily to their flight style and hunting technique. Generally, broad-winged, slow flying or hovering species, those that use passive listening or flutter-detecting hunting techniques, such as long-eared bats (*Plecotus*), mouse-eared bats (*Myotis*) and horseshoe bats (*Rhinolophus*) are particularly vulnerable to aerial predation and therefore also more intolerant to light [7]. It seems very likely that mouse-eared bats and horseshoe bats react to light installations in a similar way as the long-eared bats, and, therefore, buildings with roosts of any of these species should not be subject to external flood-lighting.

Extensive lights form barriers that restrict bats' movements and fragment their foraging grounds with potentially serious effects [26,27]. Long-eared bats generally avoid urban areas [24], presumably because of the presence of lights, and the disappearance of these and other species from lit areas and a corresponding increase in more light-exploiting species [9,28,29] typically leads to a trivialization of bat communities, where a few common species predominate [30]. The brown long-eared bat is still common in Sweden, but increasing light pollution will inevitably lead to problems for this and other light-averse bats. It is time to consider light pollution as an emerging (and escalating) problem in conservation plans that involve bats.

We do not know if the bats that disappeared from the lit churches died or survived and reproduced elsewhere. Likewise, we do not know if and how the bats that remained in lit churches were affected by the lights. It seems possible that adverse effects such as a decline in colony size or reproductive success may have occurred, but passed unnoticed by us because we did not look for them. Serious effects have been recorded in bats of other species occupying lit roosts elsewhere [17–19]. The fact that colonies sometimes remained after the installation of lights does not necessarily mean that the roosting and foraging conditions remained the same, only that continued use of the church was the preferred option for the bat colony at that time. Along the same line, it should be noted that a bat colony normally requires several exits from the roost for predator avoidance purposes [31], which means that flood-lights on parts of the building that are not currently used for exit and return may still have serious effects in the long run, if important alternative exits are blocked by the light.

In one of our churches the long-eared bat colony disappeared following installation of lights inside the roost. The lights were installed to make the internal architecture visible. In another study, a colony of Natterer's bat was ‘trapped’ inside a church attic by light installed there. They did not emerge to feed normally and were threatened by starvation, and this situation remained until the light was switched off permanently [20]. Clearly, lights inside an attic with bats must be used with great care or it may lead to disaster for the bats. Preferably, it should be avoided entirely.

Interestingly, the turnover rate of *P. auritus* colonies in churches that remained unaffected by flood-lights was zero over the 25+ years of the study, which suggests that colonies may persist continuously in the same buildings over very long periods, certainly decades and perhaps centuries, unless the conditions change drastically and force the bats to either move or die. It seems obvious that such roosts are extremely important for the bats and that forced abandonment is likely to have a strong negative effect, even if the bats manage to find an alternative roost.

Our results indicate that when bats abandoned churches, they usually did so because of light-installations. Nevertheless, it remains possible that renovations were partly responsible in four specific cases, although we have no evidence that this actually happened. Renovations and light installations are sometimes done at the same time and afterwards it may not be possible to tell which activity was the direct reason why the bats disappeared. On the other hand, although renovations obviously may cause serious disturbance in the short term, this is not necessarily important in a longer perspective, if the conditions return to normal after the renovation [24]. A speculative but possible scenario could be that bat colonies were scared away initially by the renovation work, and later, when the work was over, may either have returned to the roost or been discouraged from doing so by the flood-lights installed in the meantime.

It is indeed encouraging that the bats remained in some churches despite the installation of flood-lights. The minimum requirement from the bats' point of view seems to be that one side or one end of the church remain unlit, preferably the part that is nearest to surrounding protective tree canopies [9,32]. This tolerance from the bats' side may perhaps open up for compromising mitigation measures, where flood-lights or other lights are used on one side of the building, leaving the other in darkness for the bats to emerge and return in safety.

We are not aware of any systematic persecution of bats in Swedish churches, although it, of course, cannot be excluded that this happens occasionally, as in other buildings. However, we are confident that the light installations on the churches surveyed here were made out of ignorance about the environmental effects and without any intention to disturb or eliminate bats.

## Supplementary Material

Supplement “Age of enlightenment: long-term effects of artificial outdoor lighting on bats in churches”. Rydell et al. 2017.

## Data accessibility

The dataset supporting this article has been uploaded as electronic supplementary material.

## Permissions

The churches surveyed here were either open during the summer or were opened by the wardens on our request. We did not enter locked rooms or compartments, captured no animals and took no samples.
